# Integrated metabolomic and transcriptomic analysis of *Pogostemon cablin* shed new light on the complete biosynthesis pathway of pogostone

**DOI:** 10.3389/fpls.2025.1510184

**Published:** 2025-02-17

**Authors:** Sen Wang, Zhaoqi Zeng, Qi Zhang, Wenhua Liu, Qinjian Liu, Chong Xie, Jinlong Bei, Bingxian Chen, Aixia Zhang

**Affiliations:** ^1^ Guangdong Provincial Key Laboratory for Crop Germplasm Resources Preservation and Utilization, Agro-Biological Gene Research Center, Guangdong Academy of Agricultural Sciences, Guangzhou, China; ^2^ College of Agriculture and Biology, Zhongkai University of Agriculture and Engineering, Guangzhou, China; ^3^ College of Resources and Environment, Zhongkai University of Agriculture and Engineering, Guangzhou, China

**Keywords:** patchoulol, pogostone, biosynthesis, key enzyme, transcriptomics, patchouli, metabolomics

## Abstract

*Pogostemon cablin* (patchouli) is a well-known perennial herbaceous plant for traditional Chinese medicine, and its primary bioactive compounds are patchoulol and pogostone. The biosynthesis pathway of patchouli has been well resolved early, while the biosynthesis pathway of pogostone is still not fully resolved due to the lack of terminal enzyme directly synthesizing pogostone. Here, the present study aims to predict the terminal enzyme of pogostone biosynthesis and reconstruct its most possible complete biosynthesis, through the integrated transcriptomic and metabolomic analysis. The metabolomic and transcriptomic profiles of patchouli leaf were largely different to those of root and stem. Patchoulol analogs like patchoulene and germacrene mainly accumulated in leaf, while pogostone content was much higher in root. Based on the integrated analysis of differentially expressed genes and metabolites, we reconstructed the biosynthesis pathways of patchoulol, and predicted the most likely complete biosynthesis pathway of pogostone. Besides, we identified 29 highly-expressed genes involved in pogostone biosynthesis for the neo-octoploid genome of patchouli, and most of their expression levels were strongly correlated with pogostone content. In particular, patchouli BAHD-DCR acyltransferases (BAHD-DCRs) were phylogenetically distant from but structurally similar to the other known plant BAHD acyltransferases. Most of them possessed the conservative catalysis motif HXXXD, and the catalysis center could bind to the widely recognized substrate molecules of 4-hydroxy-6-methyl-2-pyrone and 4-methylvaleryl-CoA and product molecule of pogostone. Thus, the highly-expressed BAHD-DCRs in patchouli root were proposed to be terminal enzymes directly synthesizing pogostone. The findings here provide more supporting evidence for the medical use of patchouli whole plants, and make an important step forward fully resolving the pogostone biosynthesis pathway. The identified genes involved in pogostone biosynthesis, especially *BAHD-DCRs*, deserve further investigation and utilization in the synthetic production of pogostone.

## 1 Introduction

*Pogostemon cablin* (Blanco) Benth, also known as patchouli, is a perennial herbaceous plant of the Lamiaceae family. The shoots of patchouli have been used as traditional Chinese medicine, which has extraordinary therapy effects on digestive, pyretic, and emetic diseases (Chang et al., 2019). In Southern China, patchouli is ranked as one of the top 10 medical plants. Besides, the essential oil extracted from patchouli leaves is widely used as fragrance (Huang, 2021). The primary bioactive compounds of patchouli shoots are patchoulol and pogostone, both exhibiting outstanding antioxidant, antibacterial, anti-inflammatory properties, and aromatic odors (Ma et al., 2020). According to the relative content of these two compounds, patchouli varieties have been traditionally classified into patchoulol-type and pogostone-type (Gu et al., 2022), providing genetic resources for biological studies and breeding of patchouli.

Resolving the biosynthesis pathways of patchoulol and pogostone has been the hot-spot of biological studies on patchouli for a long time (Zhang et al., 2007). Currently, the patchoulol biosynthesis pathway has been well resolved, and the genes encoding key enzymes such as hydroxy-methyl-glutaryl-CoA reductase (*HMGR*), farnesyl diphosphate synthase (*FPPS*), and patchoulol synthase (*PTS*), have been cloned and functionally verified (Yao et al., 2021). Notably, PTS can produce over 12 sesquiterpenoids from the substrate FPP, such as patchoulol, α/β/γ-patchoulene, patchoulenol, germacrene A/D, α-guaiene, α-bulnesene, nerolidol, seychellene, and humulene (Deguerry et al., 2006; Hartwig et al., 2014). Thus, the patchoulol oil extracted from patchouli is a mixture of multiple sesquiterpenoids similar to patchoulol. Furthermore, the expression of *HMGR*, *FPPS*, and *PTS* has been found to be regulated by the microRNA *miR156*, the transcription factor R2R3-MYB, and the hormone JA response elements JAZ-MYC2 (Zhong, 2019; Zhou, 2021). Besides, the efficient synthesis of patchoulol by microbial cell factories have been successfully established in yeast (Liu et al., 2021; Tao et al., 2024).

Different from the early-resolved synthesis pathway of patchoulol, the pogostone biosynthesis pathway is still not fully resolved. Recently, Chen et al. (2021) and Wang et al. (2022) have proposed several biosynthesis pathways of pogostone, and resolved a key enzyme 2-isobutylmalate synthase (IBMS) PcIBMS1 catalyzing the synthesis of 4-methyvaleric acid through one-carbon α-ketoacid elongation pathway, and the key acyl-activating enzyme (AAE) PcAAE2 catalyzing the acylation of 4-methylvaleric acid to produce 4-methylvaleryl-CoA. The terminal enzyme catalyzing the synthesis of pogostone from 4-hydroxy-6-methyl-2-pyrone and 4-methylvaleryl-CoA is still not found, although a polyketide synthase PcPKS3 has been shown to have similar functions (Liu, 2022). In addition, the chemical synthesis of pogostone has been achieved successfully (Chen et al., 2016; Yu et al., 2018), but the reaction conditions and agents are complex and not environmentally friendly. Therefore, it is still in urgent need to fully resolve the biosynthesis pathway of pogostone.

Over the past decade, multi-omics analysis has become an effective approach to resolve the metabolism pathways of plant bioactive secondary metabolites (Alami et al., 2022). As for patchouli, transcriptomics combined with molecular biology have been used to resolve the expression of patchoulol biosynthesis genes and its regulation network (Chen, 2018; Zhong, 2019; Zhou, 2021), and the cloned biosynthesis genes of pogostone are also identified from transcriptomic data (Liu, 2022). Further insights into pogostone synthesis are not generated by the recent transcriptomic, proteomic, or metabolomic studies on patchouli, which focus on the general metabolism difference between patchoulol- and pogostone-type and sesquiterpenoid synthesis pathway (Zhang et al., 2022, 2023b), metabolism of floral development and its difference from stem and leaf (Wang et al., 2023; Zhang et al., 2023a). Besides, the genome of patchouli is an neo-octoploid (He et al., 2018, 2016; Shen et al., 2022), and the biosynthesis genes of sesquiterpenoids like patchoulol have undergone replication, expansion and diversification (He et al., 2016). Thus, it is necessary to determine the highly-expressed key copies of biosynthesis genes playing major roles in patchoulol and pogostone biosynthesis.

To resolve the complete biosynthesis pathway of pogostone and identify the highly-expressed biosynthesis genes, the present study conducted an integrated transcriptomic and metabolomic analysis of the root, stem, and leave samples of patchoulol-type patchouli. We reconstructed the biosynthesis pathway of patchoulol, and predicted the most likely complete pathway of pogostone biosynthesis by identifying the terminal enzyme directly synthesizing pogostone. Besides, we determined all the highly-expressed genes involved in the biosynthesis of patchoulol and pogostone in the octoploid genome of patchouli. The findings here provide novel insights into pogostone biosynthesis metabolism and valuable resources for the biological synthesis of other patchouli bioactive compounds.

## 2 Materials and methods

### 2.1 Plant cultivation and sample collection

A local cultivar of patchoulol-type patchouli widely used in Southern China was planted in an experimental field in May 2023, which was located at the Tianhe district of Guangzhou of Guangdong province of China. Sufficient supplies of nutrient and water were provided for the growth of patchouli plants. In October 2023, three whole plants of 5-month-old patchouli were randomly sampled, with each as one biological replication. For each patchouli plant, the root, stem, and leaf parts were separated, cleaned by RNase-free water, dried by paper, and transferred to sterile tubes. Immediately, all the nine sample tubes were frozen in liquid nitrogen for 30 min, and then stored in an ultra-low-temperature refrigerator at -80°C until further processing.

### 2.2 Transcriptome sequencing and analysis

Total RNA of patchouli root, stem, and leaf samples were extracted by the CTAB-PBIOZOL method, and dissolved in DEPC-treated water. Subsequently, the RNA sample was quantified using a Qubit fluorescence quantifier and quality-checked by a Qsep400 high throughput bio-fragment analyzer. To prepare RNA-seq libraries, mRNAs with polyA tails were enriched by the Oligo(dT) method, cleaved into small fragments, and transformed into cDNAs by reverse transcription and end repair and dA-Tailing. Then, sequencing adapter ligation, DNA magnetic bead purification and fragment selection were sequentially performed to yield a sequencing library with insert size of 250-350 bp. The nine quality-checked sequencing libraries were quantified using qPCR and pooled together and sequenced on an Illumina NovaSeq 6000 platform of Metware Biotechnology Co., Ltd. (Wuhan, China), by the mode of paired-end 150 bp.

Quality control of raw sequencing data was performed using fastp v0.23.2 (Chen et al., 2018) to remove adapter-contaminated and low-quality reads. The generated clean reads were mapped to the annotated reference genome of patchouli (Gaoch1L2016, GCA_003675935.1, genomic annotation files downloaded from Figshare 10.6084/m9.figshare.c.4100495), using HISAT v2.2.1 (Kim et al., 2015) with default settings. Novel transcript assembly from transcriptome mappings was performed using StringTie v2.1.6 (Pertea et al., 2015) with default parameters. Gene expression levels were quantified using featureCounts v2.0.3 (Liao et al., 2013) with default settings to count mapped reads and compute FPKM (fragments per kilobase per million) values.

### 2.3 Metabolome detection and analysis

#### 2.3.1 UPLC-MS/MS analysis

The root, stem, and leaf samples of patchouli for metabolomic analysis were dried using vacuum freeze-drying technology in a lyophilizer (Scientz-100F), then ground (30 Hz, 1.5 min) to powder form using a grinder (MM 400, Retsch). Next, 50 mg of sample was mixed with 1200 μL -20°C pre-cooled 70% methanolic aqueous extract (with internal standards) by vortex. After centrifugation (12000 rpm, 3 min), the supernatant was aspirated and filtered through the microporous membrane of 0.22 μm for UPLC-MS/MS analysis.

An UPLC-ESI-MS/MS system (UPLC, ExionLC™ AD; MS, Applied Biosystems 4500 Q TRAP) with the UPLC column of Agilent SB-C18 (1.8 µm, 2.1 mm * 100 mm) was used for metabolome detection. The mobile phase included pure water with 0.1% formic acid (A) and acetonitrile with 0.1% formic acid (B). Gradient program was employed for compound separation: starting 95% A, 5% B; within 9 min, linear gradient to 5% A, 95% B and kept for 1 min; within 1.1 min, linear gradient to 95% A, 5.0% B and kept for 2.9 min. The flow rate was 0.35 mL/min, the column oven was set to 40°C, and the injection volume was 4 μL. The effluent was alternatively connected to an ESI-triple quadrupole-linear ion trap (QTRAP)-MS.

The ESI source operation parameters were: source temperature 550°C; ion spray voltage (IS) 5500 V (positive ion mode)/-4500 V (negative ion mode); ion source gas I (GSI), gas II(GSII), curtain gas (CUR) set at 50, 60, and 25 psi, respectively; the collision-activated dissociation (CAD) was high. QQQ scans were acquired as MRM experiments with collision gas (nitrogen) set to medium. DP (declustering potential) and CE (collision energy) for individual MRM transitions was done with further DP and CE optimization. A specific set of MRM transitions were monitored for each period according to the metabolites eluted within this period.

#### 2.3.2 GC-MS analysis

The root, stem, and leaf samples of patchouli were ground to powder in liquid nitrogen. Immediately, 500 mg of the powder was transferred to a 20 mL head-space vial (Agilent), containing NaCl saturated solution to inhibit any enzyme reaction. The vials were sealed using crimp-top caps with TFE-silicone headspace septa (Agilent). Before analysis, each vial was placed in 60°C for 5 min, then a 120 µm DVB/CWR/PDMS fibre (Agilent) was exposed to the headspace of the sample for 15 min at 60°C.

Desorption of the VOCs (volatile organic compounds) from the fibre coating was carried out in the injection port of the GC apparatus (Model 8890; Agilent) at 250°C for 5 min in the splitless mode. The identification and quantification of VOCs was carried out using an Agilent 8890 GC coupled with an Agilent 7000E mass spectrometer, equipped with a 30 m × 0.25 mm × 0.25 μm DB-5MS (5% phenyl-polymethylsiloxane) capillary column. Helium was used as the carrier gas at the flow rate of 1.2 mL/min. The injector temperature was kept at 250°C. The oven temperature was programmed from 40°C (3.5 min), increasing to 100°C at 10°C/min, to 180°C at 7°C/min, to 280°C at 25°C/min, and hold for 5 min. Mass spectra was recorded in electron impact (EI) ionization mode at 70 eV. The quadrupole mass detector, ion source and transfer line temperatures were set at 150, 230 and 280°C, respectively. The MS selected ion monitoring (SIM) mode was used for the identification and quantification of analytes.

### 2.4 Analysis of differential expressed genes and metabolites

DEG analysis between two sample groups (for example leaf vs stem) was conducted using the R package DESeq2 v1.22.1 (Love et al., 2014; Varet et al., 2016), and the P-values were adjusted by Benjamini & Hochberg (BH) method to control false positives (Chen et al., 2017). The adjusted P-value of FDR < 0.05 and the absolute value of log_2_-transformed fold change (Log2FC) > 1 were used as thresholds to identify DEGs. The functional enrichment analysis of DEGs was performed by the hypergeometric test using the R package clusterProfiler v4.6.0 (Wu et al., 2021), based on the KEGG ortholog annotation of genes and their mappings to the KEGG pathways.

DEM analysis was conducted by the similar method as DEG analysis and the orthogonal partial least square discrimination analysis (OPLS-DA) analysis. The thresholds of VIP (variable importance in the projection) > 1 and absolute value of Log2FC > 1 were used to identify DEMs. The OPLS-DA analysis was performed using the R package MetaboAnalystR 1.0.1 (Chong and Xia, 2018), with the log2-transformed metabolite content data as input. The functional enrichment analysis of DEMs was conducted by the similar method as DEG analysis, based on the KEGG annotation of identified metabolites.

### 2.5 Reconstruction of biosynthesis pathways for important secondary metabolites

The genes and metabolites involved in the biosynthesis of sesquiterpenoids and flavonoids were identified by the KEGG annotations of DEGs and DEMs. To reconstruct the biosynthesis pathway of sesquiterpenoids and flavonoids for patchouli, the number of identified DEGs were mapped to the integrated pathways of KEGG map00900 and map00909 (sesquiterpenoid) and map00940 and map00941 (flavonoid). The full lists of DEGs and DEMs involved in sesquiterpenoid and flavonoid biosynthesis are given in **Supplementary Tables S4**, **S5**, respectively.

The complete biosynthesis pathway of pogostone has not been fully resolved. Refer to the previously proposed multiple synthesis pathways of pogostone (Chen et al., 2021; Liu, 2022; Wang et al., 2022), all the potential genes involved in pogostone biosynthesis were identified based on the functional annotation of DEGs and DEMs, and the correlations between pogostone content with the FPKM levels of these genes. Besides, the probable terminal enzyme (BAHD-DCR acyltransferase) of pogostone synthesis was predicted by phylogeny analysis and molecular docking. The multiple sequence alignment of patchouli BAHD-DCR and other plant BAHD acyltransferases was conducted using ClustalO v1.2.4 (Sievers et al., 2011) with default settings, and the maximum-likelihood phylogeny tree was built using RAxML v8.2.10 (Stamatakis, 2014) with parameters “-m PROTGAMMALGX -f a -N 1000”. The structures of patchouli BAHD-DCR enzyme and its complex with substrate (4-hydroxy-6-methyl-2-pyrone and 4-methylvaleryl-CoA) and product (pogostone) molecules were predicted using AlphaFold3 (Abramson et al., 2024) with default settings.

### 2.6 Expression validation of selected biosynthesis genes

For each enzyme of the biosynthesis pathway of sesquiterpenoids, flavonoids, and pogostone, there are multiple encoding genes and the DEG with the highest expression levels in root, stem, or leaf was selected for expression level validation. In total, 19 genes were selected and their specific PCR primers were designed by NCBI Primer-BLAST (**Supplementary Table S6**). The real-time reverse transcription quantitative PCR (RT-qPCR) method was used to determine the expression levels of these genes.

Total RNA was extracted using a modified CTAB method. The first strand of the reverse-transcribed cDNA was synthesized using the Monad first-strand cDNA Synthesis Kit. RT-qPCR experiments were performed by the ABI7500 quantitative PCR instrument. The tubulin gene of patchouli (*PcTubulin3*) was selected as internal reference gene. The relative expression of each gene was calculated using the 2^-ΔΔCt^ method (Livak and Schmittgen, 2001).

### 2.7 Statistical analysis and plotting

Principal component analysis (PCA) of the gene expression profiles of different samples was performed using the function prcomp of R v4.1.2, with the Z-score scaled FPKM values as input. The PCA ordination plots of gene expression profiles, violin and box plots of gene expression levels and metabolite contents, volcano plots of DEGs and DEMs, bubble, bar and lollipop plots of KEGG pathway enrichment of DEGs and DEMs, and pie charts of metabolomic composition were drawn using the R package ggplot2. The Pearson correlations among the metabolomic profiles of all samples were visualized as heatmaps using the R package corrplot v0.92. and the heatmap showing metabolomic profiles was drawn using the R package ComplexHeatmap v2.9.4 (Gu, 2022; Gu et al., 2016).

The differences of gene expression level and metabolite content among root, stem, and leaf were determined by least significant difference (LSD) test at P-value < 0.05. The phylogeny tree of BAHD acyltransferases was drawn using FigTree v1.4.4, and the visualization of predicted structures of patchouli BAHD-DCR enzyme and its complex with substrate and product molecules was conducted using PyMOL v2.5.5. Drawing of metabolic pathways and grouping of separate figures were completed in Adobe Illustrator 2021.

## 3 Results

### 3.1 Transcriptomic and metabolomic profiling of patchouli whole plant

**Figure 1:**
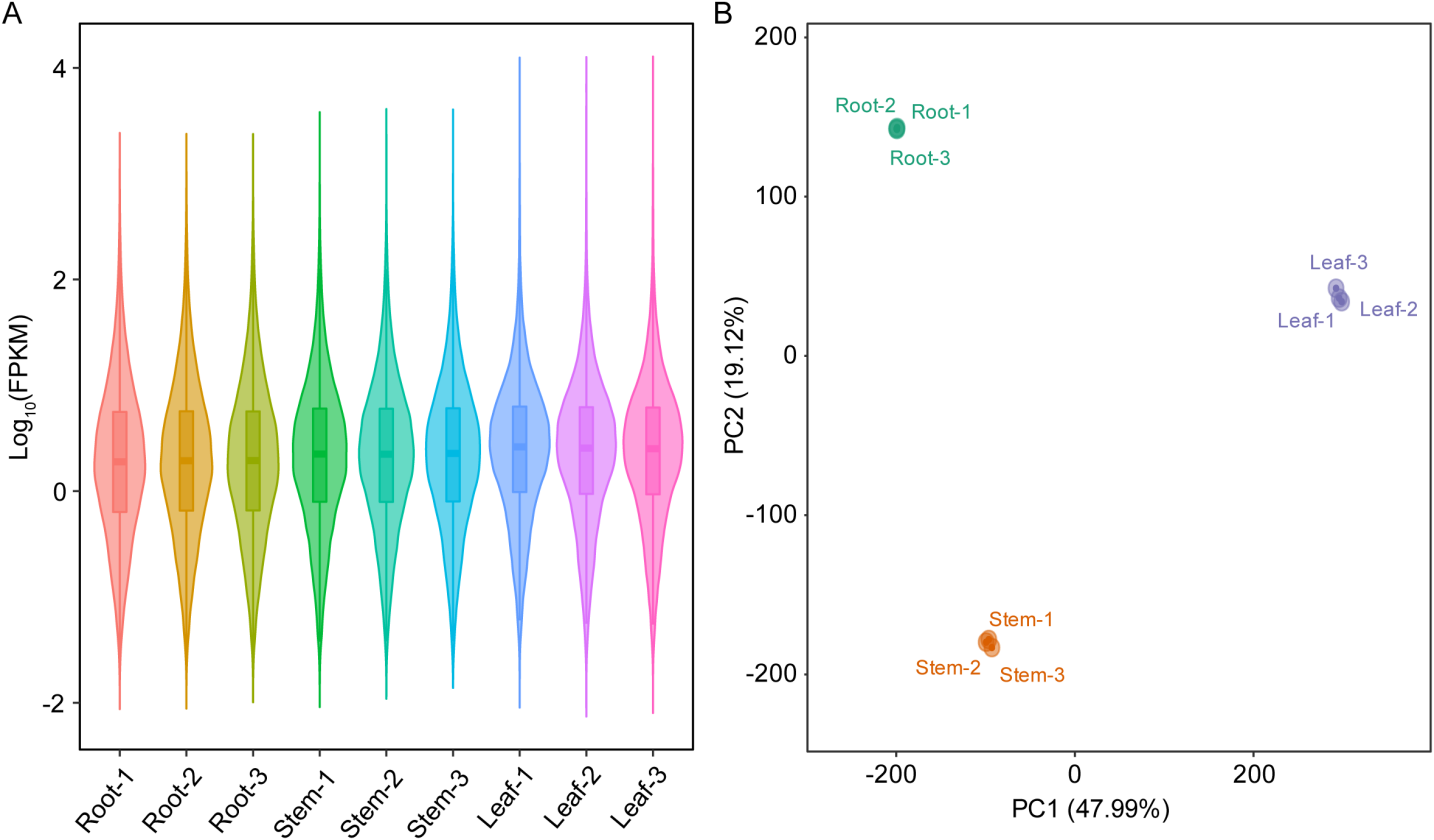
Gene expression profiles of root, stem, and leaf of patchouli. **(A)** Violin and boxplot of the log_10_-transormed fragments per kilobase per million (FPKM) values in root, stem, and leaf samples. The width of violin is proportional to the number of genes, and the lower, middle, and upper horizontal lines of box refer to 25th, median (50th), and 75th percentiles, respectively. **(B)** Ordination of samples in the first two principal components (PCs), based on the principal component analysis of gene expression matrix.

The transcriptome sequencing of root, stem and leaf samples of patchouli generated a total of 49.53 Gb clean reads (**Supplementary Table S1**), with average mapping rate of 93.94%. Besides, 10,194 novel transcripts were assembled from the transcriptomic mappings, generating a new reference gene set of 121,044 transcripts for patchouli transcriptome (**Supplementary Table S2**). The expression levels (FPKM) of most transcripts ranged from 0.1 to 100 (**Figure 1A**), and there were large differences among the gene expression profiles of root, stem, and leaf (**Figure 1B**). Overall, the transcriptomic profiles of root and stem were more similar, as indicated by the much larger number of DEGs in leaf vs stem (16,789 up- and 16,590 down-regulated) and root vs leaf (18,813 up- and 20,759 down-regulated) group than that in root vs stem (6,846 up- and 8,342 down-regulated) group (**Table 1**; **Supplementary Figures S1A–C**).

**Table 1:** Number of differentially expressed genes (DEGs) among different parts of *P. cablin*.

Comparison group	Up regulated DEGs	Down regulated DEGs	Up regulated DEMs	Down regulated DEMs
Leaf vs Stem	16,789	16,590	1,058	625
Root vs Leaf	18,813	20,759	734	1,298
Root vs Stem	6,846	8,342	594	934

DEGs (DEMs) were identified by FPKM (metabolite content) fold change > 2 (up regulated) or < 0.5 (down regulated), and FDR (false discovery rate) P-value < 0.05 in each comparison group.

**Figure 2:**
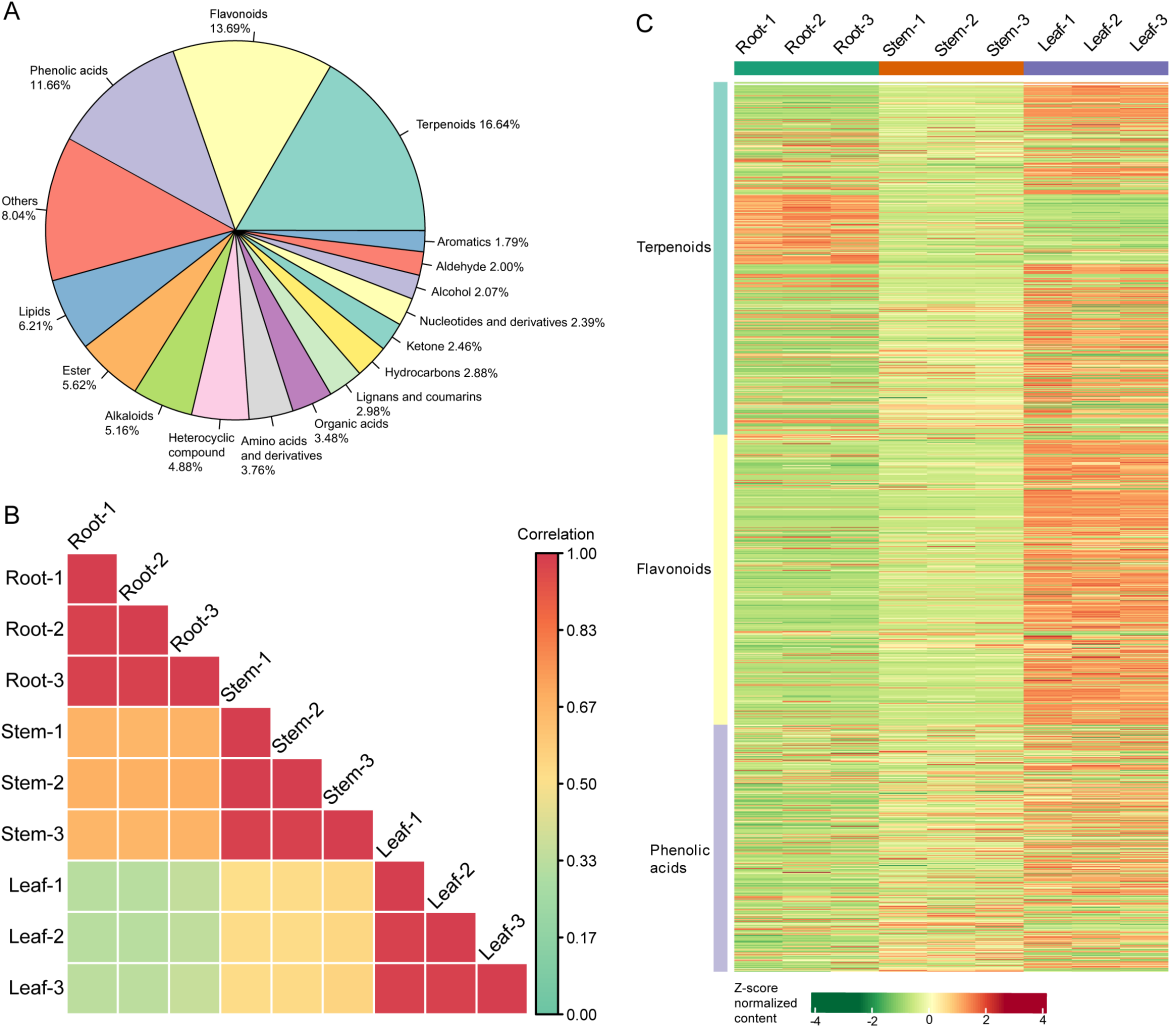
Metabolomic profiles of patchouli root, stem, and leaf. **(A)** The classification and composition of all the detected metabolites. The radians of pies are proportional to the percentages of the corresponding type of compounds, which are calculated by the number of compounds. **(B)** Heatmap showing the Pearson correlations among the metabolomic profiles of nine samples. The color of each square is proportional to the correlation coefficient between the corresponding two samples. **(C)** Heatmap showing the relative contents of terpenoid, flavonoid, and phenolic acid metabolites in nine samples. The color of each rectangle is proportional to the Z-score normalized content of the corresponding metabolite.

Metabolomics analysis detected a total of 2,848 metabolites in the root, stem, and leaf samples of patchouli (**Supplementary Table S3**). The metabolomic profile was mainly composed of terpenoids, flavonoids, phenolic acids, lipids, esters, and alkaloids (**Figure 2A**), with the content levels varied between 10^2^ and 10^8^ (**Supplementary Figure S2A**). Besides, the correlations between the metabolomic profiles of root and those of stem samples were higher than those between leaf and root and between leaf and stem samples (**Figure 2B**). Furthermore, patchouli leaf samples contained higher contents of most terpenoids, flavonoids, and phenolic acids, while root samples contained higher contents of minor terpenoids and lipids (**Figure 2C**).

### 3.2 Pathway enrichment of DEGs and DEMs

**Figure 3:**
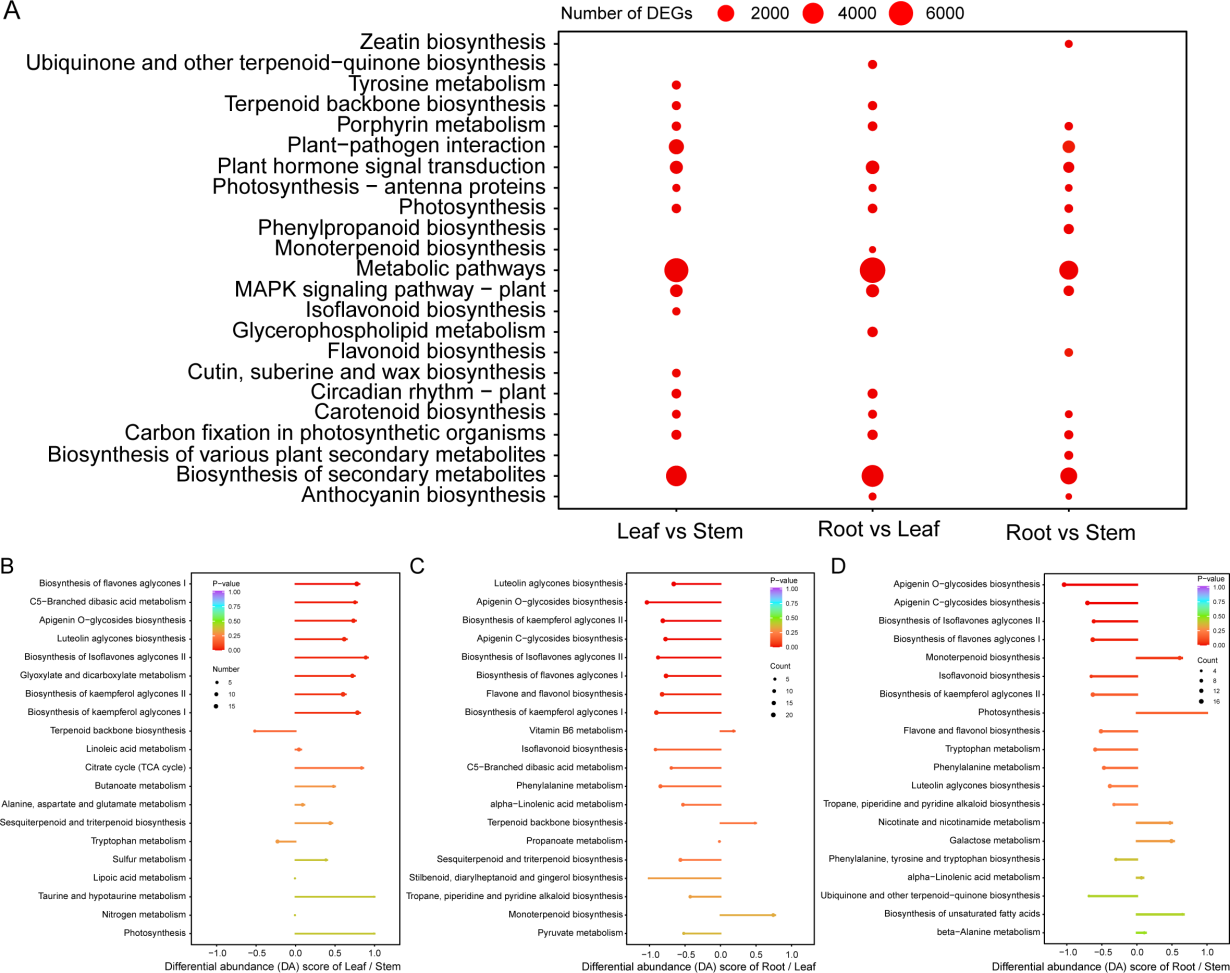
Metabolic pathway enrichment analysis of differentially expressed genes (DEGs) and metabolites (DEMs). **(A)** KEGG pathway enrichment of DEGs of leaf vs stem, root vs leaf, and root vs stem. The size of each dot is proportional to the number of DEGs in the corresponding group. **(B-D)** Lollipop plot of differential abundance (DA) score of the functional enrichment of DEMs in different KEGG pathways for the leaf vs stem, root vs leaf, and root vs stem group, respectively. The size of each dot and stick is proportional to the corresponding P-value, and the size of each dot is proportional to the number of DEMs.

The functional enrichment analysis of DEGs showed that a considerable part of DEGs participated in the biosynthesis of secondary metabolites, such as terpenoid backbone, monoterpenoid, carotenoid, zeatin, flavonoid, isoflavonoid, anthocyanin, ubiquinone, etc. (**Figure 3A**; **Supplementary Figures S3A–C**). The functional enrichment analysis results of DEMs were similar to that of DEGs. There were 1058, 734, and 594 up-regulated DEMs and 625, 1298, and 934 down-regulated DEMs in the leaf vs stem, root vs leaf, and root vs stem groups, respectively (**Table 1**; **Supplementary Figures S4A–C**), and nearly all of these DEMs are involved in the biosynthesis of secondary metabolites, especially flavones, isoflavones, and their aglycones (**Figures 3B–D**). Notably, the up-regulated DEMs in patchouli leaf were mainly involved in the biosynthesis of apigenin, luteolin, quercetin and its glycosides, sesquiterpenes, and triterpenes (**Figures 3B–D**). The large number of DEGs and DEMs between leaf and root (or stem) related with secondary metabolites provide the foundation for elucidating the synthesis pathways of important bioactive compounds in patchouli.

### 3.3 Biosynthesis pathway reconstruction of important bioactive metabolites

The most abundant (content > 10^8^) secondary metabolites in patchouli were sesquiterpenoids like patchoulene, caryophyllene, and germacrene D, etc., and flavonoids like glycosides and glucuronides of apigenin, luteolin, etc. (**Supplementary Figure S5A**; **Supplementary Table S5**). These metabolites accumulated to much higher contents in patchouli leaf tissues, while the content of pogostone was much higher in patchouli root tissues (**Supplementary Figure S5A**), corresponding to the investigated patchoulol-type cultivar in the present research. The biosynthesis pathways of patchoulol and flavonoids for patchouli (**Supplementary Figures S5B, C**) were reconstructed based on the functional mappings of DEGs and DEMs to the well-established KEGG pathways of sesquiterpenoids and flavonoids (**Supplementary Table S4**). For the octoploid genome of patchouli, each biosynthesis gene had multiple copies, and we generated 2 novel transcripts involved in patchoulol biosynthesis and 22 novel transcripts involved in flavonoid biosynthesis (**Supplementary Table S4**). In the patchoulol biosynthesis pathway, the isoprene synthesis genes of 1-deoxy-D-xylulose 5-phosphate (DXP) pathway and sesquiterpenoid synthesis genes (*FDPS/GGPS*, *GERD/PTS*) were mainly upregulated in leaf (**Supplementary Figure S5C**), consistent with the higher content of sesquiterpenoids in patchouli leaf. Similarly, the flavone synthesis genes (*F3H*, *FLS*, *FSII*, etc.) are also mainly upregulated in leaf (**Supplementary Figure S5B**), consistent with the trend of flavonoid content. Besides, RT-qPCR was used to verify the expression levels of 8 genes involving sesquiterpenoid synthesis and 7 genes involving flavonoid synthesis (**Supplementary Figure S6**).

**Figure 4:**
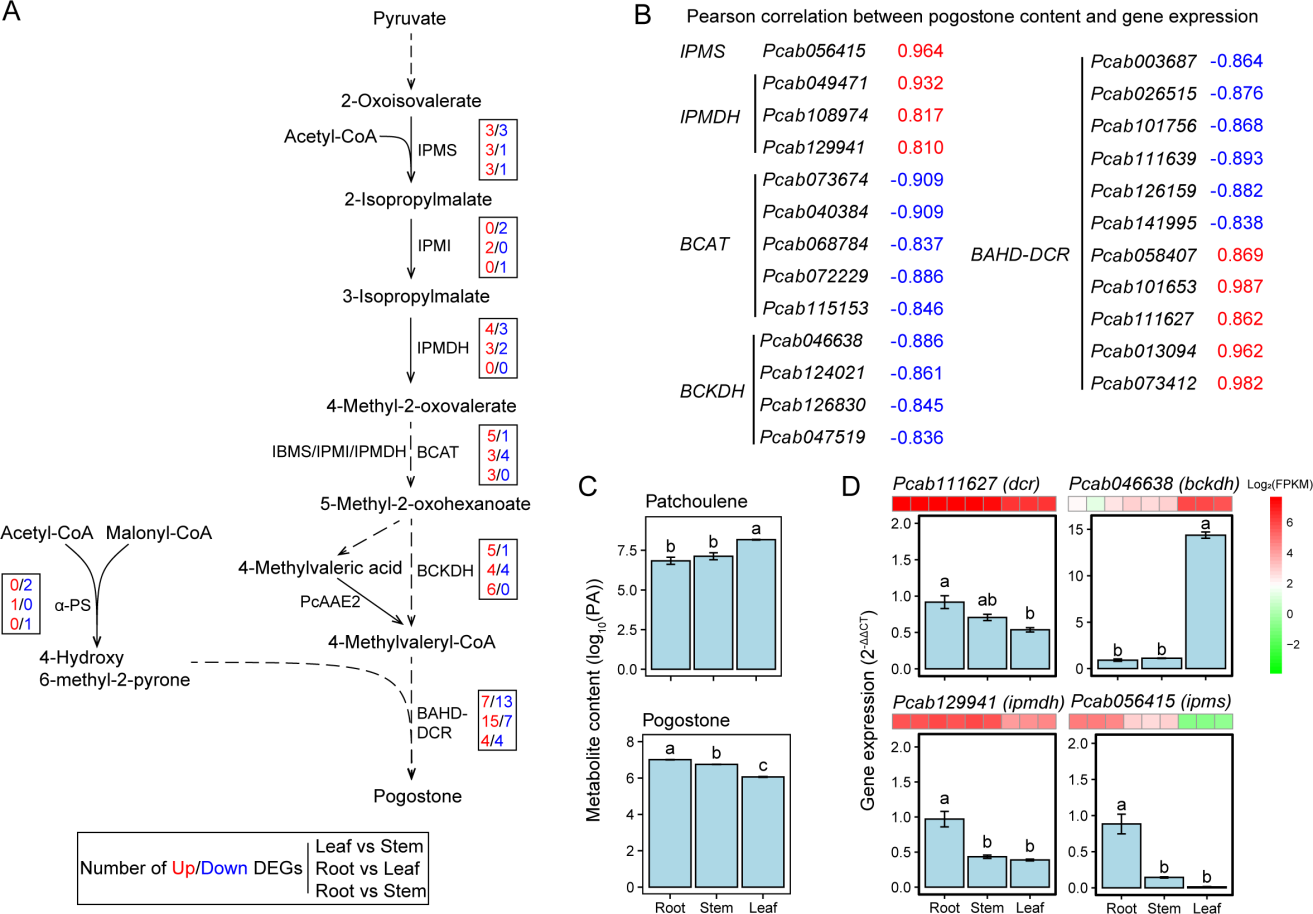
Identification of candidate genes involved in the biosynthesis pathway of pogostone. **(A)** Mapping of differentially expressed genes (DEGs) in the predicted pathway of pogostone biosynthesis. In each box, the numbers of up- and down-regulated DEGs are highlighted in red and blue color, respectively, and are presented in the order of leaf vs stem, root vs leaf, and root vs stem group. Dashed arrow lines refer to the predicted biosynthesis steps and enzymes. **(B)** Pearson correlation coefficients between pogostone content and the expression levels (FPKMs) of candidate genes involved in pogostone biosynthesis. **(C)** Log10-transformed contents of patchoulene and pogostone in patchouli root, stem, and leaf. **(D)** Expression levels (2^-ΔΔCt^) of selected candidate genes involved in pogostone biosynthesis by real-time reverse transcription quantitative PCR (RT-qPCR). For each gene, its transcriptome-based expression levels (FPKMs) are presented as a small heatmap above the corresponding bar plot. Different lowercase letters on bars indicate significant differences by LSD test at P-value < 0.05.

The biosynthesis pathway of pogostone has not been fully resolved, so we predicted the most likely biosynthetic genes and pathways for pogostone in patchouli (**Figure 4A**), refer to the previously proposed pogostone biosynthesis pathways (Chen et al., 2021; Wang et al., 2022). In particular, we identified the highly-expressed (FPKM > 10 in root, stem, or leaf) gene copies involved in pogostone biosynthesis, including 1 *IPMS* (*isopropylmalate synthase*), 1 *IPMI* (*isopropylmalate isomerase*), 3 *IPMDHs* (*isopropylmalate dehydrogenase*), 5 *BCATs* (*branched-chain aminotransferase*), 4 *BCKDHs* (*branched-chain keto acid dehydrogenase*), and 15 *BAHD-DCRs* (*BAHD-DCR acyltransferase*) (**Table 2**). The expressions of *IPMS* and *IPMDH* were strongly and positively (r > 0.8) with the content of pogostone, while the expressions of *BCAT* and *BCKDH* were strongly and negatively (r < -0.8) with the content of pogostone (**Figures 4B, C**). Besides, we also used RT-qPCR to verify the expression levels of 4 selected genes, and the results were consistent with the trends of FPKM levels (**Figure 4D**). Notably, for the predicted terminal enzyme of pogostone biosynthesis BAHD-DCR, the expression levels of 5 encoding genes were positively correlated and 6 genes were negatively correlated with pogostone content (**Figures 4B, C**). Thus, it is necessary to determine the key members of *BAHD-DCR* playing major roles in the biosynthesis of pogostone in patchouli root tissue.

**Table 2:** Highly-expressed candidate genes involved in pogostone biosynthesis.

Enzyme	Gene/Transcript ID	Average expression level (FPKM)		
		Root	Stem	Leaf
IPMS	*Pcab056415*	25.10	6.94	0.60
IPMI	*novel.14793*	5.49	21.07	11.25
IPMDH	*Pcab049471*	15.88	12.43	5.91
	*Pcab108974*	18.44	16.44	7.14
	*Pcab129941*	53.77	56.17	20.08
BCAT	*Pcab073674*	2.17	4.18	18.05
	*Pcab040384*	2.86	4.30	13.40
	*Pcab068784*	6.62	6.10	18.93
	*Pcab072229*	6.06	7.12	21.85
	*Pcab115153*	4.75	4.70	10.75
BCKDH	*Pcab046638*	3.94	7.17	56.96
	*Pcab124021*	0.69	0.67	14.11
	*Pcab013285*	26.72	14.97	25.96
	*Pcab047519*	8.31	7.37	27.02
BAHD-DCR	*Pcab003687*	0.07	0.14	14.97
	*Pcab011155*	10.38	37.53	4.06
	*Pcab026515*	0.14	0.48	10.38
	*Pcab016738*	94.73	172.35	5.18
	*Pcab101756*	0.09	0.37	17.59
	*Pcab058407*	14.12	13.37	0.00
	*Pcab111639*	0.63	1.55	10.10
	*Pcab126159*	0.59	1.43	16.89
	*Pcab067341*	122.41	200.15	11.97
	*Pcab141995*	0.90	0.70	11.40
	*Pcab101653*	11.95	6.74	0.16
	*Pcab111627*	181.63	180.51	84.76
	*Pcab013094*	10.01	7.30	0.20
	*Pcab143490*	7.24	11.54	0.26
	*Pcab073412*	39.73	25.29	0.24

Highly-expressed genes refer to those with FPKM > 10 in root, stem, or leaf tissue.

### 3.4 Predicting the key candidate terminal enzyme of pogostone biosynthesis pathway

Among the 15 predicted *BAHD-DCR* genes encoding terminal enzyme of pogostone biosynthesis, *Pcab016738*, *Pcab067341*, and *Pcab111627* showed the highest expression levels in patchouli root and stem tissues (**Table 2**). The AlphaFold3 predicted protein structure of Pcab111627 was largely different from the structures of other 14 BAHD-DCRs which all have conserved characteristics of known BAHD acyltransferases (**Supplementary Figure S7**), indicating it as not a typical acyltransferase. Thus, Pcab067341 was selected as the representative of patchouli BAHD-DCR acyltransferases for investigation of its potential catalysis ability to synthesize pogostone from 4-hydroxy-6-methyl-2-pyrone and 4-methylvaleryl-CoA.

**Figure 5:**
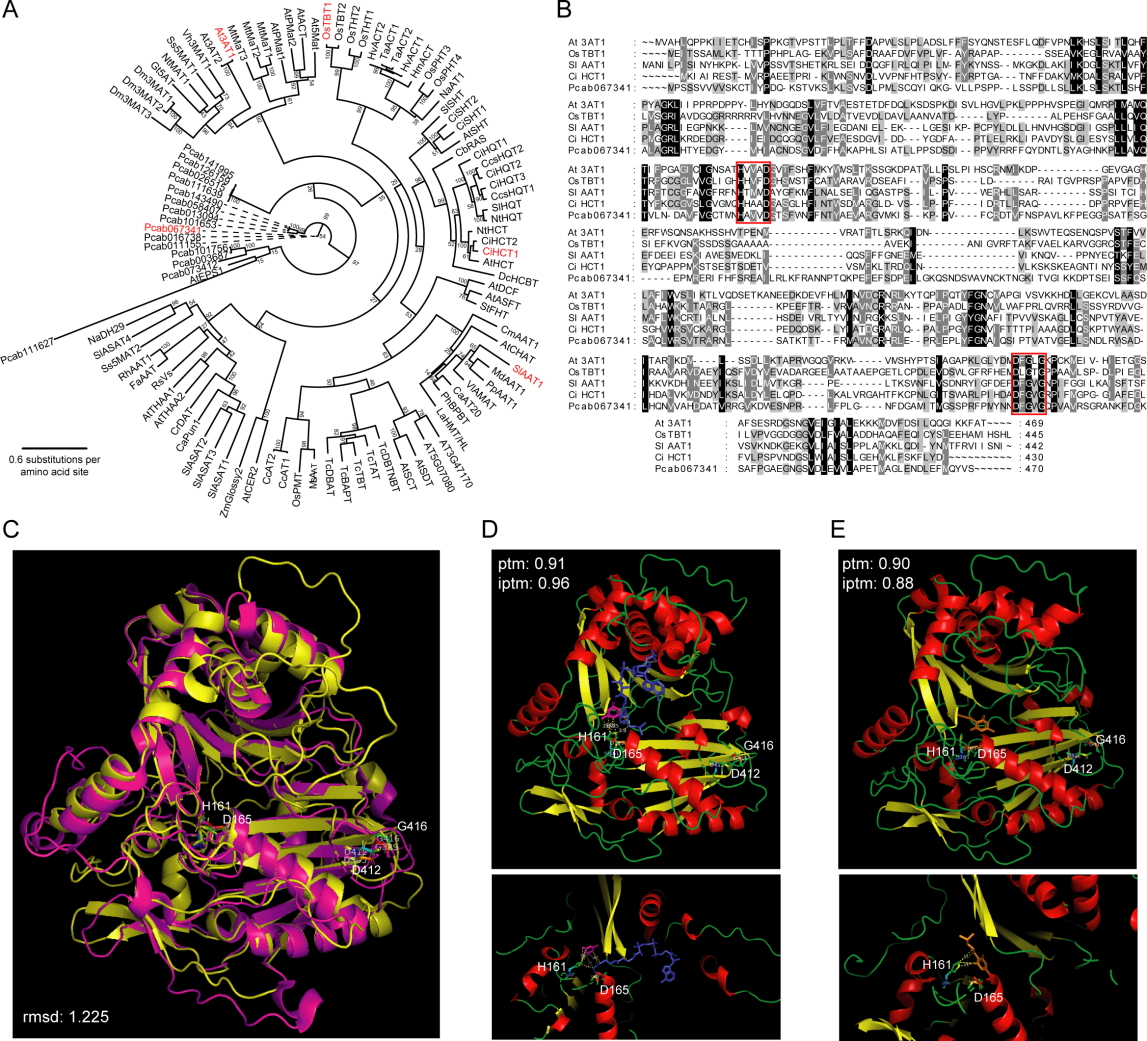
Phylogenic and structural analysis of predicted terminal enzymes for pogostone biosynthesis. **(A)** Phylogeny tree of 101 BAHD acyltransferase members from 35 species (15 BAHD-DCR genes from patchouli), built on the multiple sequence alignments (MSA) of these proteins. Numbers at internal nodes refer to bootstrap support values of the corresponding clade. **(B)** MSA of five representative BAHD enzymes (At3AT1, OsTBT1, SlAAT1, CiHCT1, and patchouli BAHD-DCR Pcab067341), with the catalysis (HXXXD) and binding (DFGWG) motifs highlighted by red rectangles. **(C)** Structural alignment of patchouli BAHD-DCR enzyme (yellow, predicted by AlphaFold3) to barley BAHD enzyme HvACT (magentas, PDB: 7CYS), and the key residues of motifs are shown as sticks (H161, D165, D412, G416). **(D, E)** Predicted complex structure of patchouli BAHD-DCR enzyme with the substrates 4-hydroxy-6-methyl-2-pyrone (magentas) and 4-methylvaleryl-CoA (blue) and the product pogostone (orange), respectively. Upper and lower panels show the global view of the whole complex and detailed view of catalysis center. Key residues are shown as sticks and their potential contacts with substrate and product molecules are marked by dashed yellow lines.

Phylogeny analysis based on multiple sequence alignment showed that patchouli BAHD-DCRs were distant from the known BAHD acyltransferases from other plant species (**Figure 5A**). But the protein structures of these acyltransferases are highly conserved, and Pcab067341 possessed the typical catalysis motif HXXXD and binding motif DFGWG as the other 4 known BAHD acyltransferases did (**Figure 5B**). The predicted structure of patchouli Pcab067341 was highly similar to the experimentally resolved structure of barley HvACT, with RMSD value as low as 1.225 and large overlaps in the α-helixes and β-sheets (**Figure 5C**). Furthermore, the AlphaFold3 predicted binding complex structure of patchouli BAHD-DCR enzyme Pcab067341 with the substrate and product molecules of pogostone synthesis showed that, 4-hydroxy-6-methyl-2-pyrone, 4-methylvaleryl-CoA and pogostone could bind to the catalysis center HXXXD with high confidence (ptm > 0.9 and iptm > 0.88) (**Figures 5D, E**). Both the acyl acceptor 4-hydroxy-6-methyl-2-pyrone and the acyl donor 4-methylvaleryl-CoA located very near to the key residue of acyl transfer reaction H161 of the HXXXD motif of Pcab067341, and the same case was also observed for product molecule pogostone (**Figures 5D, E**). These results indicate that Pcab067341, as the representative of patchouli BAHD-DCR acyltransferase, can have the catalysis ability to transfer 4-methylvaleryl to 4-hydroxy-6-methyl-2-pyrone and produce pogostone.

## 4 Discussion

### 4.1 Predicting the terminal synthase of pogostone to resolve its complete biosynthesis pathway

The present study predicts the key candidate pogostone synthase BAHD-DCR, and reconstructs the most likely complete biosynthesis pathway of pogostone (**Figures 4**, **5**). Pogostone has been the medical quality marker metabolite of patchouli, with stronger bioactivities than patchoulol (Yi et al., 2013), while its biosynthesis pathway is still not fully resolved. Although the chemical synthesis of pogostone have been successfully established (Chen et al., 2016; Yu et al., 2018), and several possible biosynthesis pathways have been proposed for pogostone (Chen et al., 2021; Liu, 2022), we still do not know the exact enzyme directly synthesizing pogostone. The final step of pogostone is an acylation reaction, in which 4-methylvaleryl is transferred from 4-methylvaleryl CoA to the backbone of pogostone, 4-hydroxy-6-methyl-2-pyrone. The key enzymes involved in the biosynthesis 4-methylvaleryl CoA, PcIBMS1 and PcAAE2, have been well resolved (Chen et al., 2021; Wang et al., 2022). Here, we predict the patchoulol BAHD-DCR acyltransferases to be the terminal enzyme directly synthesizing pogostone, making an important step forward the complete biosynthesis pathway of pogostone in patchouli.

Plant BAHD acyltransferases catalyze the acyl transfer from fatty acyls (acetyl-CoA, malonyl CoA, succinyl-CoA, etc.) and aromatic acyls (cinnamoyl-CoA, caffeoyl-CoA, coumaroyl-CoA, etc.), to alcohols (flavonoids, terpenoids, etc.) and amines (polyamines, alkaloids, etc.) (Xu et al., 2023a). BAHDs from different species often have different preferences in acyl donor and acceptor substrates. For the patchouli BAHD-DCRs identified here, they are phylogenetically distant from but structurally close to other known plant BAHDs, and have the typical catalysis motif HXXXD and binding motif DFGWG and the catalysis center can bind to the molecules of 4-methylvaleryl CoA, 4-hydroxy-6-methyl-2-pyrone, and pogostone with high confidence (**Figure 5**). Thus, the patchouli BAHD-DCR acyltransferase can have the catalysis ability to use 4-methylvaleryl CoA as acyl donor and 4-hydroxy-6-methyl-2-pyrone as acyl acceptor to synthesize pogostone. It should be noted that, the exact functions of patchouli BAHD-DCRs still needs to be verified by further biochemical studies.

### 4.2 Expression differentiation of patchoulol and pogostone biosynthesis genes in octoploid patchouli

In the present research, we provide all the actively-expressed transcripts for each enzyme of patchoulol and pogostone biosynthesis, and the downstream genes often show expression differentiation between patchouli root and leaf (**Table 2**; **Supplementary Table S4**; **Figure 4**; **Supplementary Figure S5**). For example, of the 12 highly-expressed *FDPS/GGPS* genes involving patchoulol precursor synthesis, 5 show high expression in root while 7 show high expression mainly in leaf, and similar cases are also observed for the *BAHD-DCR* genes involving pogostone synthesis (**Supplementary Table S4**). This can be the result of gene expansion and differentiation during the complex evolution history of the neo-octoploid genome of patchouli (Shen et al., 2022). In future studies on the metabolism of patchoulol and pogostone, more attention should be paid to the highly and specifically expressed genes in specific tissues.

The biosynthesis of the C5 precursors of patchoulol, isopentenyl-PP and dimethylallyl-PP, have two pathways namely the cytoplasm-located mevalonate (MVA) pathway and plastid-located DXP pathway. It can be expected that the genes of DXP pathway are mainly expressed in leaf tissues while the genes of MVA pathway are mainly expressed in root tissues, consistent with the distribution of up-regulated DEGs in patchouli root and leaf in the present study (**Supplementary Figure S5**). Similar results are also observed previous studies on the expression and regulation of patchoulol synthesis genes, in different organs or across various growth stages (Chen, 2018; Zhang et al., 2023a, 2022; Zhou, 2021). Thus, both patchouli root and leaf tissues can contain abundant C5 units of terpenoids, while the encoding genes of terminal enzyme of patchoulol GERD/PTS mainly expressed in leaf (**Supplementary Figure S5**), leading to the synthesis and accumulation of patchoulol analogs in leaf tissues.

### 4.3 Combining multi-omics with in-silico enzyme analysis help resolve the biosynthesis pathway of more bioactive metabolites for patchouli

For the patchoulol-type patchouli investigated here, we find that the major patchoulol analogs in leaf tissues are patchoulene and germacrene (**Supplementary Figure S5A**). This can be due to the catalysis diversity of patchoulol synthase PTS, which has been reported to synthesize more than 12 sesquiterpenoid compounds similar to patchoulol (Deguerry et al., 2006). Also, the patchoulol oil extracted from patchouli shoot parts is a mixture of multiple sesquiterpenoid metabolites (Xu et al., 2023b). Notably, the root tissues of the patchoulol-type patchouli tested here contain relatively large amount of pogostone (**Figure 4C**; **Supplementary Figure S5A**), indicating the potential use of patchouli roots for extracting pogostone, except for the traditional use of patchouli shoots as Chinese medicine. The opposite change trends of pogostone and patchoulol contents across different parts and growth stages are also observed in previous studies (Zhang et al., 2022, 2023b), which can be ascribed to the competition for common precursors. Furthermore, pyruvate and acetyl-CoA are the starting substrate of both pogostone and patchoulol biosynthesis, and also the likely competitive precursors, which needs further investigation in future studies.

This study proposes the BAHD-DCR acyltransferase to be the terminal enzyme directly synthesizing pogostone and reconstructs the most likely complete biosynthesis pathway (**Figures 4**, **5**) for patchoulol-type patchouli, through integrating transcriptomic and metabolomic analysis of different plant parts and the in-silico structural analysis of key enzymes. Whether these findings are also true for pogostone-type patchouli needs further investigation, since there may be genetic and metabolic variations between both chemical types concerning pogostone metabolism. In previous studies on patchouli, the integrated metabolomic, transcriptomic, and proteomic analysis has been used to investigate the molecular mechanisms of the formation of patchoulol- and pogostone-type patchouli (Zhang et al., 2023a, 2022), and the regulation network of flower development (Wang et al., 2023; Zhang et al., 2023b). However, these studies did not pay much attention to the biosynthesis genes of pogostone, possibly due to its not fully resolved synthesis pathway. In future studies on the biosynthesis of other bioactive compounds in patchouli, the combined analysis of different tissues and growth stages of both chemical types is recommended to generate more complete knowledge for one synthesis pathway, and also more in-depth insights into the mechanism of key steps and enzymes.

## 5 Conclusion

Through the integrated transcriptomic and metabolomic analysis of patchouli whole plants and the in-silico structural analysis of enzyme proteins, the present study reconstructs the biosynthesis pathways of sesquiterpenoids (patchoulol, patchoulene, etc.) and flavonoids, and predicts the most likely complete synthesis pathway of pogostone. Besides, the genes encoding downstream enzymes of patchoulol and pogostone biosynthesis have expression differentiation between root and leaf tissues, and the highly-expressed genes playing major roles are identified. In particular, we predict more than 10 patchouli BAHD-DCR enzymes to be the candidate terminal enzyme directly synthesizing pogostone. Patchouli BAHD-DCR acyltransferases are phylogenetically distant from but structurally similar to the known plant BAHD acyltransferases, and possess the typical catalysis motif HXXXD and can bind to the substrate and product molecules. Furthermore, we identify the major BAHD-DCRs involving pogostone synthesis in patchouli root tissues, which deserves further investigation in future studies. Collectively, the findings here provide more supports for the medical use of whole patchouli plants, and valuable resources and approaches for the in-depth studies on the biosynthesis of other bioactive metabolites as well as their synthetic production.

## Data Availability

The datasets presented in this study can be found in online repositories. The names of the repository/repositories and accession number(s) can be found in the article/**Supplementary Material**.

## Supplementary material

Supplementary Table 1 Statistics of transcriptome sequencing and mapping.

Supplementary Table 2 Expression and annotation of all genes and transcripts from patchouli transcriptome data.

Supplementary Table 3 Identification and quantification of all metabolites by GC-MS and UPLC-MS analysis.

Supplementary Table 4 Candidate differentially expressed genes involved in the biosynthesis of bioactive compounds.

Supplementary Table 5 Candidate differentially expressed metabolites of the metabolism of bioactive compounds.

Supplementary Table 6 PCR primers of key genes involved in the biosynthesis of bioactive compounds.

## References

[B1] Abramson J. Adler J. Dunger J. Evans R. Green T. Pritzel A.. (2024). Accurate structure prediction of biomolecular interactions with AlphaFold 3. Nature 630, 493–500. doi: 10.1038/s41586-024-07487-w 38718835 PMC11168924

[B2] Alami M. M. Ouyang Z. Zhang Y. Shu S. Yang G. Mei Z.. (2022). The current developments in medicinal plant genomics enabled the diversification of secondary metabolites’ biosynthesis. Int. J. Mol. Sci. 23, 15932. doi: 10.3390/ijms232415932 36555572 PMC9781956

[B3] Chang Y. Junyan G. Weizhe W. Puwang L. Ziming Y. Xinyu K.. (2019). The efficacy and application progress of *Pogostemon cablin* . Chin. J. Trop. Agric. 39, 68–74. doi: 10.12008/j.issn.1009-2196.2019.12.011

[B4] Chen H. (2018). The transcriptome sequencing of *Pogostemon cablin* leaves and analysis of *FPS, PTS, HMGR* gene differential expression (Haikou: Hainan University), 72.

[B5] Chen H. Zhou J. Liu Y. Zhang Z. Zhan Y. Su Z.. (2016). Synthesis of pogostone by one-step. J. Asian Natural Products Res. 19, 172–175. doi: 10.1080/10286020.2016.1184253 27243631

[B6] Chen J. Liu L. Wang Y. Li Z. Wang G. Kraus G. A.. (2021). Characterization of a cytosolic acyl-activating enzyme catalyzing the formation of 4-methylvaleryl-CoA for pogostone biosynthesis in *Pogostemon cablin* . Plant Cell Physiol. 62, 1556–1571. doi: 10.1093/pcp/pcab111 34255851 PMC8643619

[B7] Chen S. Zhou Y. Chen Y. Gu J. (2018). Fastp: an ultra-fast all-in-one FASTQ preprocessor. Bioinformatics 34, 884–890. doi: 10.1093/bioinformatics/bty560 30423086 PMC6129281

[B8] Chen S. Y. Feng Z. Yi X. (2017). A general introduction to adjustment for multiple comparisons. J. Thorac. Dis. 9, 1725–1729. doi: 10.21037/jtd.2017.05.34 28740688 PMC5506159

[B9] Chong J. Xia J. (2018). MetaboAnalystR: an R package for flexible and reproducible analysis of metabolomics data. Bioinformatics 34, 4313–4314. doi: 10.1093/bioinformatics/bty528 29955821 PMC6289126

[B10] Deguerry F. Pastore L. Wu S. Clark A. Chappell J. Schalk M. (2006). The diverse sesquiterpene profile of patchouli, *Pogostemon cablin*, is correlated with a limited number of sesquiterpene synthases. Arch. Biochem. Biophysics 454, 123–136. doi: 10.1016/j.abb.2006.08.006 16970904

[B11] Gu Z. (2022). Complex heatmap visualization. iMeta 1, e43. doi: 10.1002/imt2.v1.3 38868715 PMC10989952

[B12] Gu Z. Eils R. Schlesner M. (2016). Complex heatmaps reveal patterns and correlations in multidimensional genomic data. Bioinformatics 32, 2847–2849. doi: 10.1093/bioinformatics/btw313 27207943

[B13] Gu Y. Mei Y. Xu S. Sun M. Zhou F. Li J.. (2022). Research progress on germplasm resources and cultivation techniques of *Pogostemon cablin* . Chin. J. Trop. Crops 43, 1595–1603. doi: 10.3969/j.issn.1000-2561.2022.08.008

[B14] Hartwig S. Frister T. Alemdar S. Li Z. Krings U. Berger R. G.. (2014). Expression, purification and activity assay of a patchoulol synthase cDNA variant fused to thioredoxin in *Escherichia coli* . Protein Expression Purification 97, 61–71. doi: 10.1016/j.pep.2014.02.003 24576659

[B15] He Y. Peng F. Deng C. Xiong L. Huang Z. Zhang R.. (2018). Building an octaploid genome and transcriptome of the medicinal plant *Pogostemon cablin* from Lamiales. Sci. Data 5, 180274. doi: 10.1038/sdata.2018.274 30532075 PMC6289116

[B16] He Y. Xiao H. Deng C. Xiong L. Nie H. Peng C. (2016). Survey of the genome of *Pogostemon cablin* provides insights into its evolutionary history and sesquiterpenoid biosynthesis. Sci. Rep. 6, 26405. doi: 10.1038/srep26405 27198881 PMC4873823

[B17] Huang B. (2021). Study on the germplasm resource identification, volatiole oil extraction and biological activity of medical plant patchouli Vol. 81 (Yichun: Yichun University).

[B18] Kim D. Langmead B. Salzberg S. L. (2015). HISAT: a fast spliced aligner with low memory requirements. Nat. Methods 12, 357–360. doi: 10.1038/nmeth.3317 25751142 PMC4655817

[B19] Liao Y. Smyth G. K. Shi W. (2013). FeatureCounts: an efficient general purpose program for assigning sequence reads to genomic features. Bioinformatics 30, 923–930. doi: 10.1093/bioinformatics/btt656 24227677

[B20] Liu L. (2022). Identification of key genes that catalyze pogostone biosynthesis (Chongqing: Chongqing University), 86.

[B21] Liu M. Lin Y. C. Guo J. J. Du M. M. Tao X. Gao B.. (2021). High-Level production of sesquiterpene patchoulol in *Saccharomyces cerevisiae* . ACS synthetic Biol. 10, 158–172. doi: 10.1021/acssynbio.0c00521 33395273

[B22] Livak K. J. Schmittgen T. D. (2001). Analysis of relative gene expression data using real-time quantitative PCR and the 2^-ΔΔCt^ Method. Methods 25, 402–408. doi: 10.1006/meth.2001.1262 11846609

[B23] Love M. I. Huber W. Anders S. (2014). Moderated estimation of fold change and dispersion for RNA-seq data with DESeq2. Genome Biol. 15, 550. doi: 10.1186/s13059-014-0550-8 25516281 PMC4302049

[B24] Ma C. Peng C. Li X. Xiong L. Zhou Q. (2020). Research progress on chemical composition and pharmacological activities of patchouli. J. Chengdu Univ. TCM 43, 72–80. doi: 10.13593/j.cnki.51-1501/r.2020.01.072

[B25] Pertea M. Pertea G. M. Antonescu C. M. Chang T.-C. Mendell J. T. Salzberg S. L. (2015). StringTie enables improved reconstruction of a transcriptome from RNA-seq reads. Nat. Biotechnol. 33, 290–295. doi: 10.1038/nbt.3122 25690850 PMC4643835

[B26] Shen Y. Li W. Zeng Y. Li Z. Chen Y. Zhang J.. (2022). Chromosome-level and haplotype-resolved genome provides insight into the tetraploid hybrid origin of patchouli. Nat. Commun. 13, 3511. doi: 10.1038/s41467-022-31121-w 35717499 PMC9206139

[B27] Sievers F. Wilm A. Dineen D. Gibson T. J. Karplus K. Li W.. (2011). Fast, scalable generation of high-quality protein multiple sequence alignments using Clustal Omega. Mol. Syst. Biol. 7, 539. doi: 10.1038/msb.2011.75 21988835 PMC3261699

[B28] Stamatakis A. (2014). RAxML version 8: a tool for phylogenetic analysis and post-analysis of large phylogenies. Bioinformatics 30, 1312–1313. doi: 10.1093/bioinformatics/btu033 24451623 PMC3998144

[B29] Tao Q. Du G. Chen J. Zhang J. Peng Z. (2024). Metabolic engineering for ffficient synthesis of patchoulol in *Saccharomyces cerevisiae* . Fermentation 10, 211. doi: 10.3390/fermentation10040211

[B30] Varet H. Brillet-Guéguen L. Coppée J.-Y. Dillies M.-A. (2016). SARTools: a DESeq2- and EdgeR-based R pipeline for comprehensive differential analysis of RNA-Seq data. PloS One 11, e0157022. doi: 10.1371/journal.pone.0157022 27280887 PMC4900645

[B31] Wang C. Wang Y. Chen J. Liu L. Yang M. Li Z.. (2022). Synthesis of 4-methylvaleric acid, a precursor of pogostone, involves a 2-isobutylmalate synthase related to 2-isopropylmalate synthase of leucine biosynthesis. New Phytol. 235, 1129–1145. doi: 10.1111/nph.v235.3 35485988

[B32] Wang X. Zhong L. Zou X. Gong L. Zhuang J. Zhang D.. (2023). GC-MS and UHPLC-QTOFMS-assisted identification of the differential metabolites and metabolic pathways in key tissues of *Pogostemon cablin* . Front. Plant Sci. 14, 1098280. doi: 10.3389/fpls.2023.1098280 36923120 PMC10009150

[B33] Wu T. Hu E. Xu S. Chen M. Guo P. Dai Z.. (2021). ClusterProfiler 4.0: A universal enrichment tool for interpreting omics data. Innovation (Cambridge (Mass)) 2, 100141. doi: 10.1016/j.xinn.2021.100141 34557778 PMC8454663

[B34] Xu W. Li X. Peng T. Wang B. Wu H. Huang Y. (2023b). Chemical composition and metabolomis analysis of non⁃targeted metabolites of three patchouli oils. Acad. J. Shanghai Univ. Traditional Chin. Med. 37, 19–30. doi: 10.16306/j.1008⁃861x.2023.05.003

[B35] Xu D. Wang Z. Zhuang W. Wang T. Xie Y. (2023a). Family characteristics, phylogenetic reconstruction, and potential applications of the plant BAHD acyltransferase family. Front. Plant Sci. 14, 1218914. doi: 10.3389/fpls.2023.1218914 37868312 PMC10585174

[B36] Yao Y. He M. Li Y. Xia J. Yan H. Zhang H. (2021). Biosynthesis and metabolism regulation of terpenoids in *Pogostemon cablin*: a review. China J. Chin. Materia Med. 46, 5560–5567. doi: 10.19540/j.cnki.cjcmm.20210722.102 34951207

[B37] Yi Y. He J. Su J. Kong S. Su J. Li Y.. (2013). Synthesis and antimicrobial evaluation of pogostone and its analogues. Fitoterapia 84, 135–139. doi: 10.1016/j.fitote.2012.11.005 23160088

[B38] Yu J. Landberg J. Shavarebi F. Bilanchone V. Okerlund A. Wanninayake U.. (2018). Bioengineering triacetic acid lactone production in *Yarrowia lipolytica* for pogostone synthesis. Biotechnol. Bioengineering 115, 2383–2388. doi: 10.1002/bit.v115.9 PMC6855914 29777591

[B39] Zhang H. Deng W. Lu C. He M. Yan H. (2022). SMRT sequencing of full-length transcriptome and gene expression analysis in two chemical types of *Pogostemon cablin* (Blanco) Benth. PeerJ 10, e12940. doi: 10.7717/peerj.12940 35223208 PMC8877398

[B40] Zhang C. Liu X. Liu Y. Yu J. Yao G. Yang H.. (2023a). An integrated transcriptome and metabolome analysis reveals the gene network regulating flower development in *Pogostemon cablin* . Front. Plant Sci. 14, 1201486. doi: 10.3389/fpls.2023.1201486 37457333 PMC10340533

[B41] Zhang H. Ou X. Chen W. Zeng Q. Yan Y. He M.. (2023b). Comparative physicochemical, hormonal, transcriptomic and proteomic analyses provide new insights into the formation mechanism of two chemotypes of *Pogostemon cablin* . PloS One 18, e0290402. doi: 10.1371/journal.pone.0290402 37738267 PMC10516424

[B42] Zhang C. Sun H. Zhongjun G. Zengrong Z. (2007). Plant terpenoid natural metabolism pathways and their synthases. Plant Physiol. Commun. 43, 779–786.

[B43] Zhong L. (2019). Metabolomes of muli-tissues of patchouli and preliminary study on metabolites and proteins responded to MeJA-induced in leaves (Guangzhou: Guangzhou University of Chinese Medicine), 80.

[B44] Zhou X. (2021). Analysis of the transcriptome of patchouli leaves in response to exogenous hormones and identify of regulators (Guangzhou: Guangzhou University of Chinese Medicine), 69.

