# Supplementary material for: Integrated metabolomic and transcriptomic analysis of Pogostemon cablin shed new light on the complete biosynthesis pathway of pogostone
Source: Front Plant Sci. 2025 Feb 17;16:1510184. doi: 10.3389/fpls.2025.1510184 (PMC11872920; doi:10.3389/fpls.2025.1510184)
Supplement: Supplementary file 7 [file DataSheet1.docx]

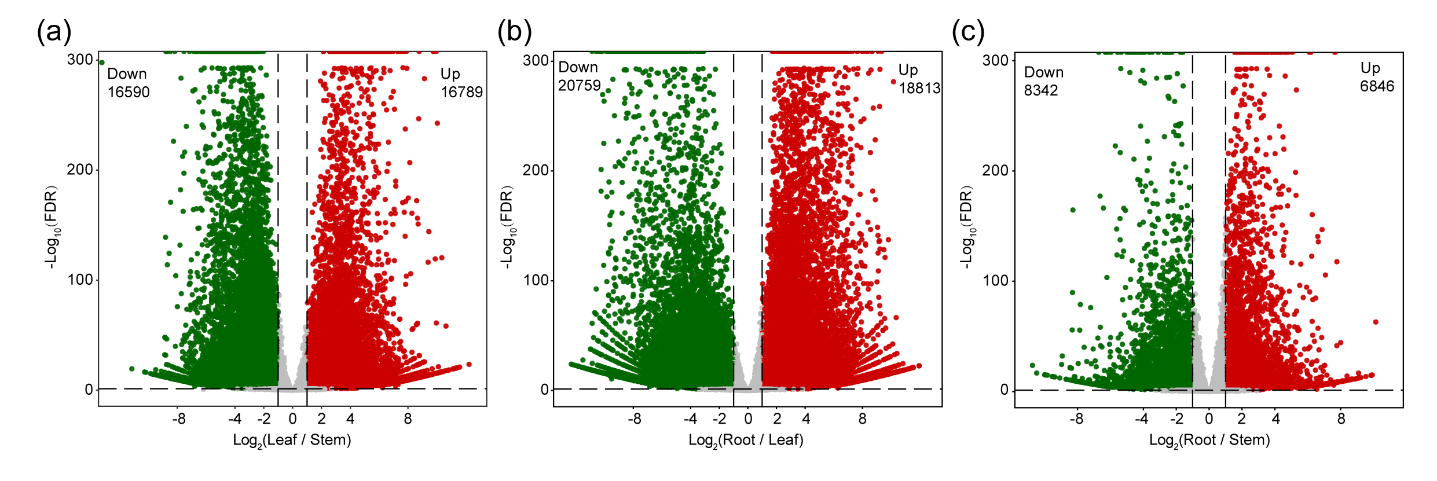


**Figure S1** Differentially expressed genes (DEGs) in root, stem, and leaf of patchouli. **(a)**, **(b)**, and **(c)** Volcano plot of DEGs by log_2_-transformed fold change and log10-transformed false discovery rate (FDR) P-value in leaf vs stem, root vs leaf, root vs stem group, respectively. Up and down regulated DEGs are highlighted in red and green color, respectively.


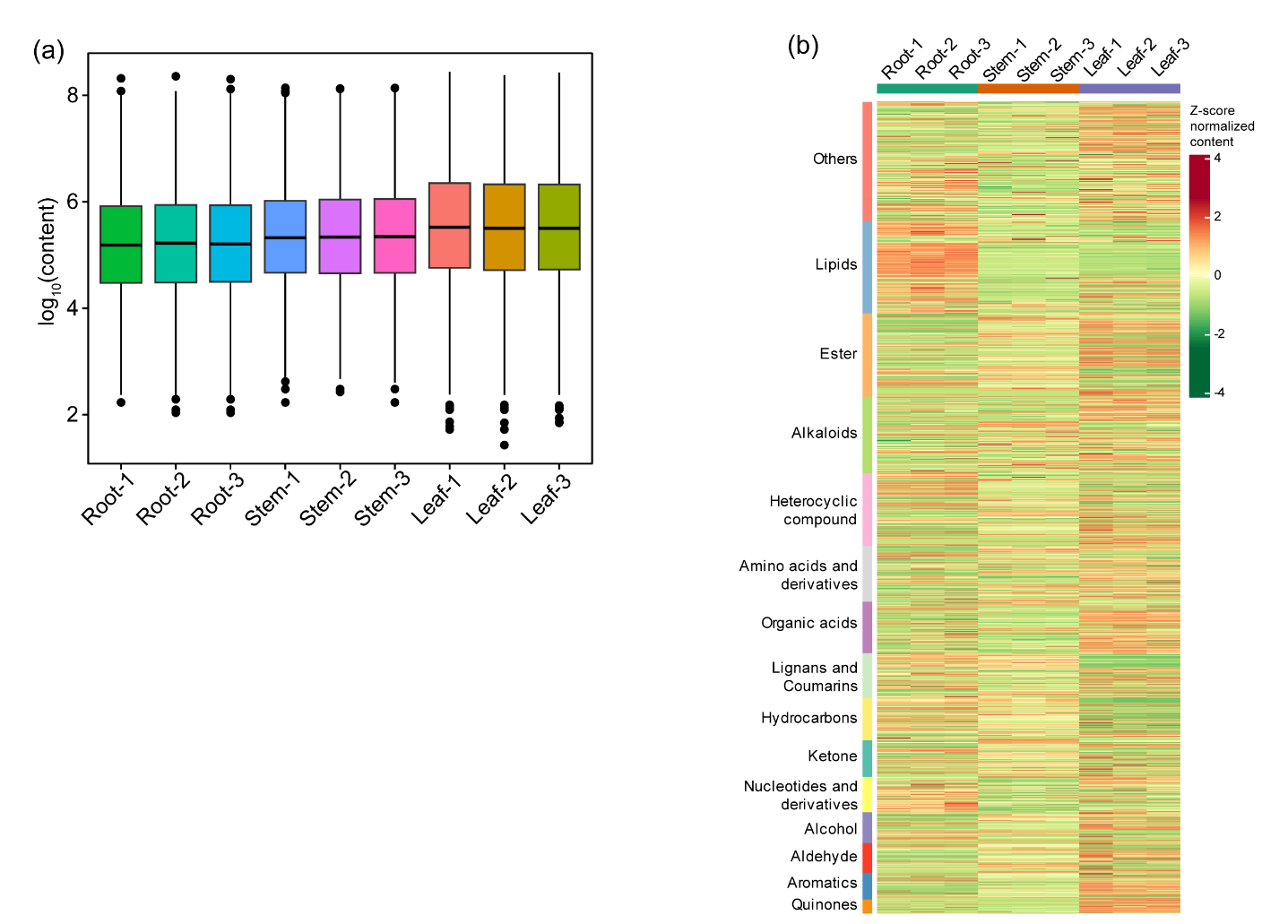


**Figure S2** Metabolomic profiles of patchouli root, stem, and leaf. **(a)** Boxplot of log_10_-transformed contents (areas of chromatographic peaks) of all metabolites in patchouli root, stem, and leaf samples. The lower, middle, and upper horizontal lines of box refer to 25th, median (50th), and 75th percentiles, respectively, and outliers are shown as solid black circles. **(b)** Heatmap showing the relative contents of other secondary metabolites (except for those shown in **Figure 2c**) in nine samples. The color of each rectangle is proportional to the Z-score normalized content of the corresponding metabolite.


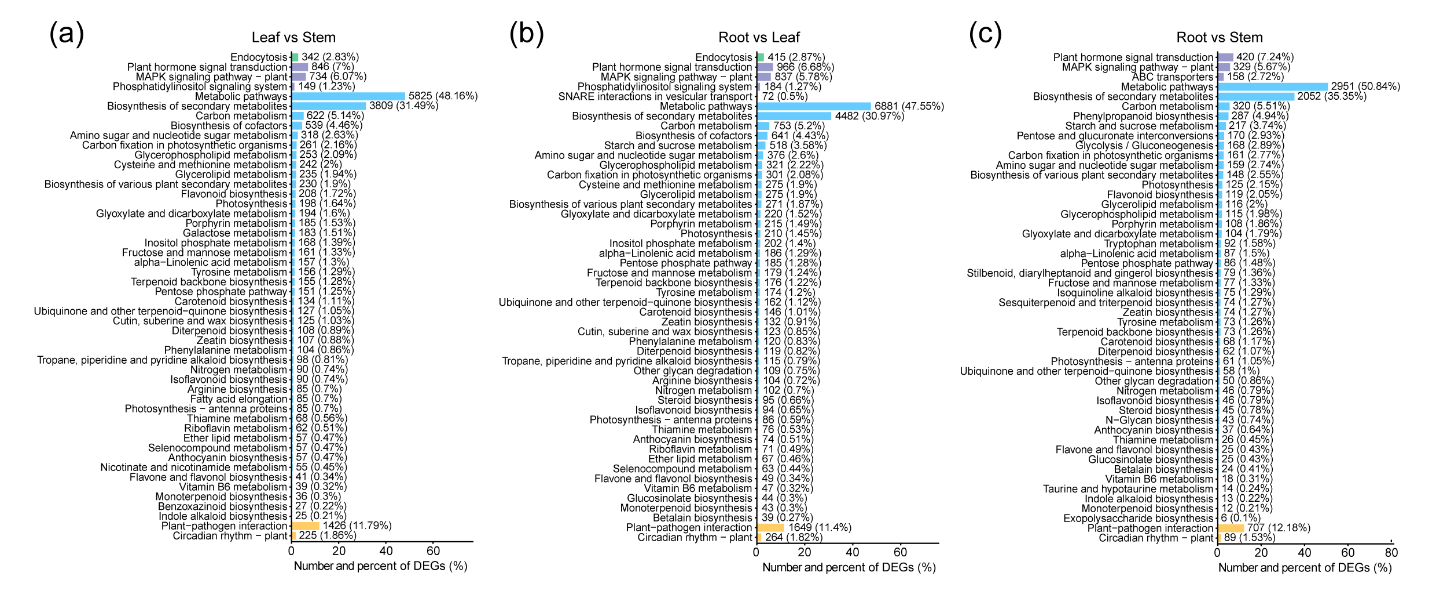


**Figure S3** Metabolic pathway enrichment analysis of differentially expressed genes (DEGs). **(a)**, **(b)** and **(c)** Number and percent of DEGs enriched in different KEGG pathways for the comparison group of leaf vs stem, root vs leaf, and root vs stem, respectively.


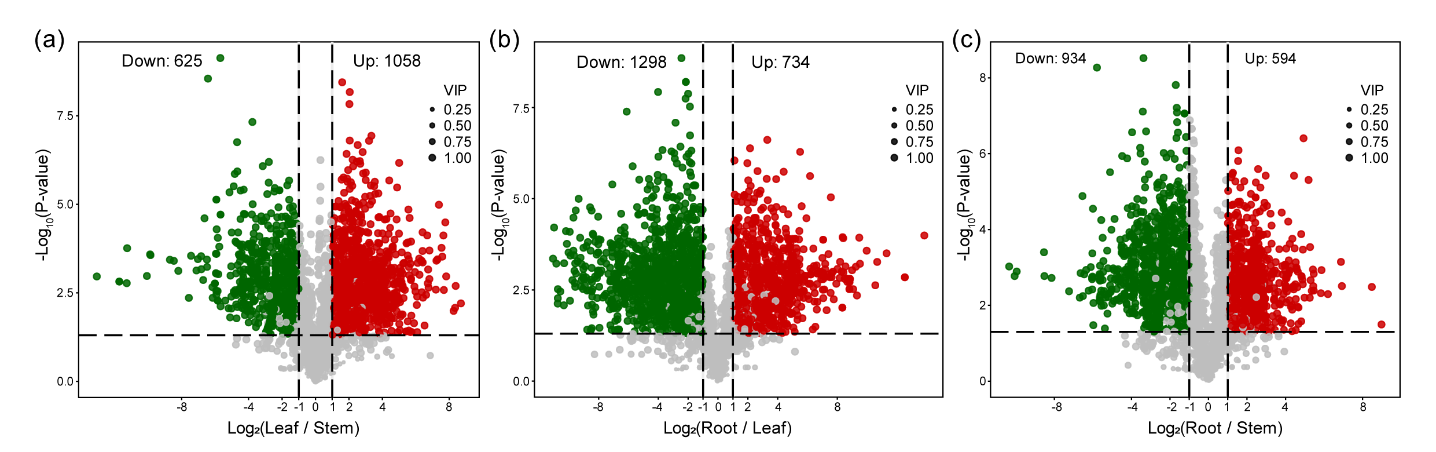


**Figure S4** Differentially expressed metabolites (DEMs) in different comparison groups of patchouli. **(a)**, **(b)**, and **(c)** Volcano plot of the DEMs by log_2_-transformed fold change and -log_10_-transformed P-value in the leaf vs stem, root vs leaf, and root vs stem groups, respectively. Up- and Down-regulated DEMs are highlighted in red and green color, and the size of each dot is proportional to the variable importance in the projection (VIP) of orthogonal partial least square discrimination analysis (OPLS-DA).


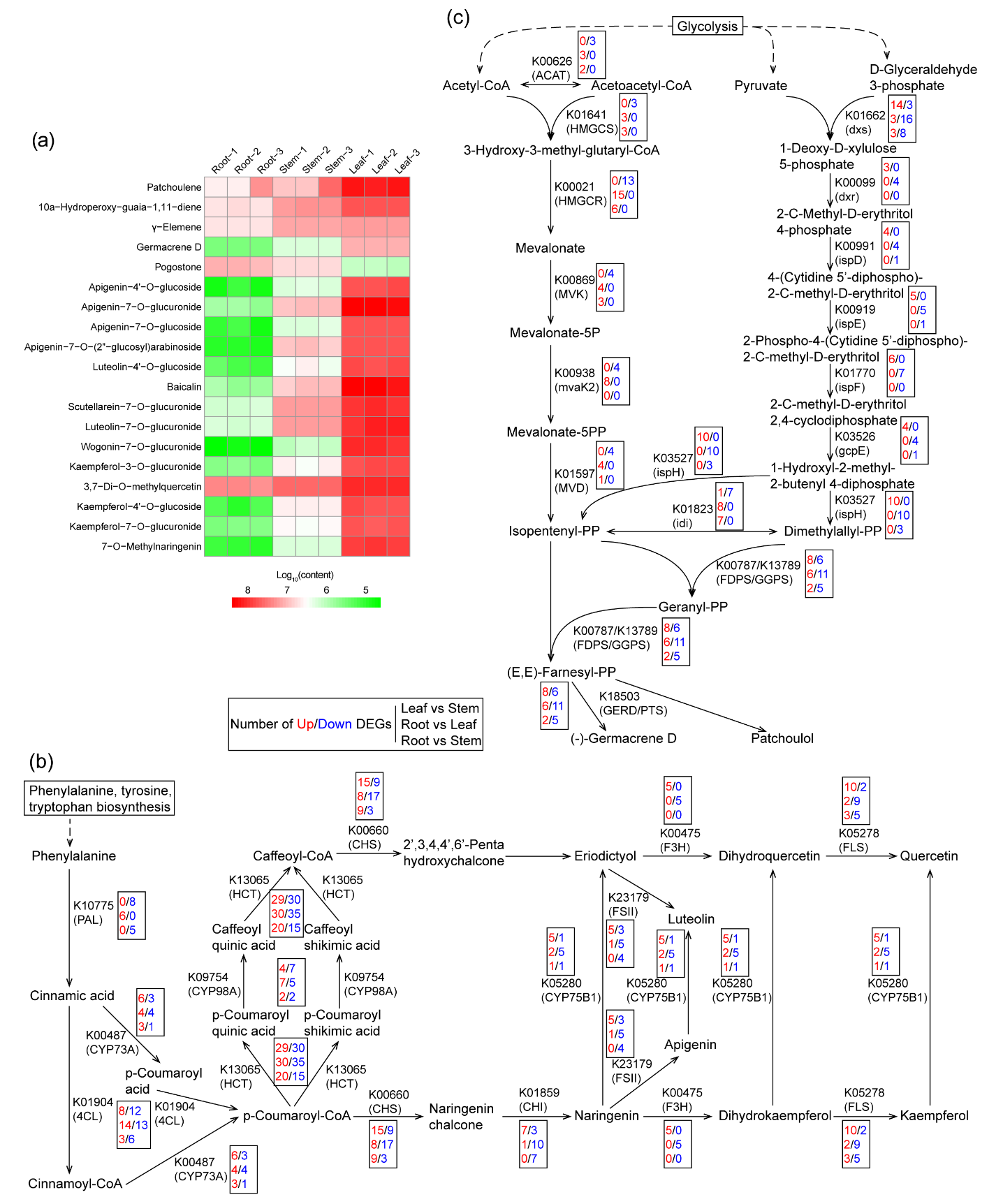


**Figure S5** Mapping of differentially expressed genes (DEGs) in the biosynthesis pathway of important secondary metabolites for patchouli. **(a)** Heatmap showing the contents of major sesquiterpenoids, flavonoids, and pogostone in patchouli root, stem, and leaf samples. The color of each square is proportional to the log_10_-transformed chromatographic peak area of the corresponding compound. **(b)** and **(c)** Mapping of DEGs in the biosynthesis pathways of flavonoids and sesquiterpenoids. In each box, the numbers of up- and down-regulated DEGs are highlighted in red and blue color, respectively, and are presented in the order of leaf vs stem, root vs leaf, and root vs stem group.


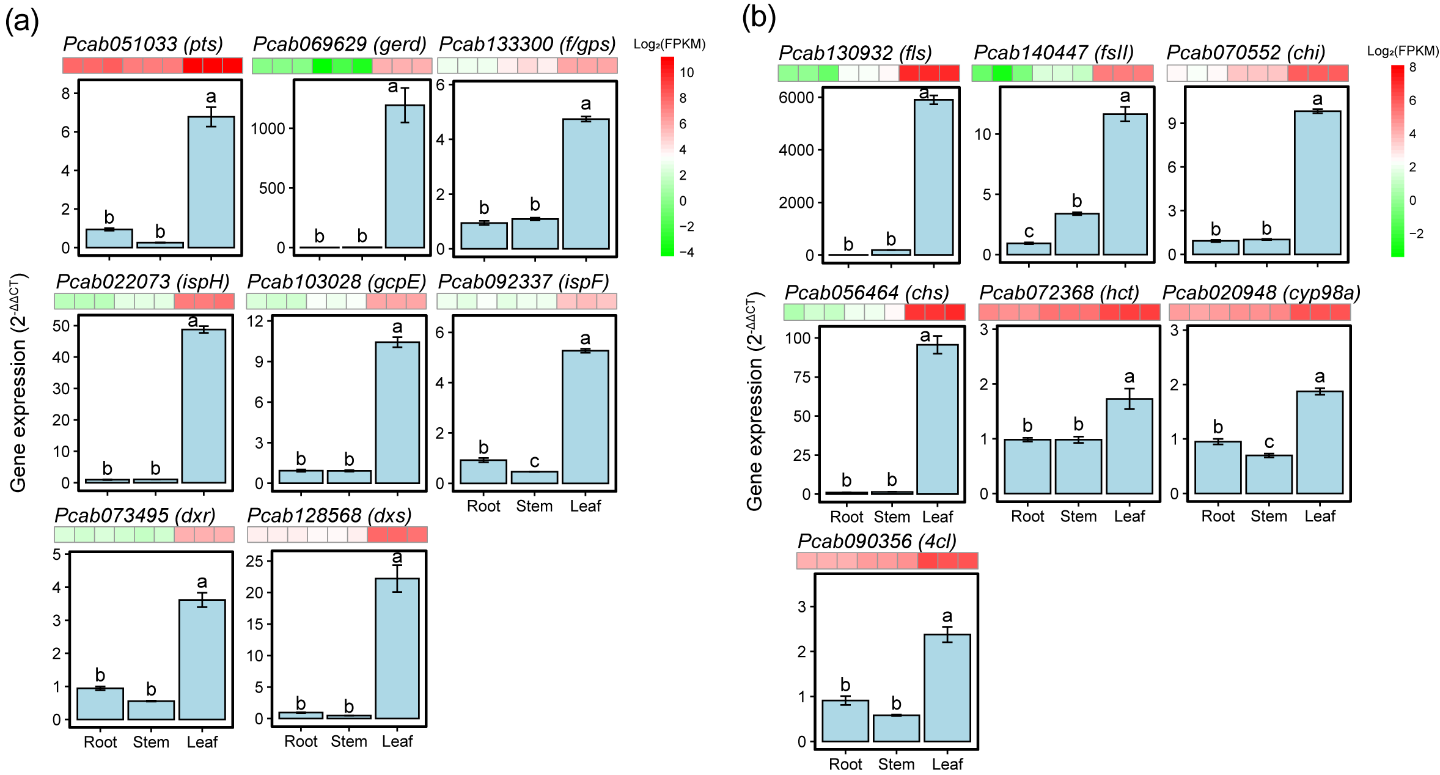


**Figure S6** Expression verification of key and highly-expressed biosynthesis genes of important bioactive metabolites for patchouli. **(a)** and **(b)** Expression levels (2^-ΔΔCT^) of selected candidate genes involved in the biosynthesis of sesquiterpenoids and flavonoids by real-time reverse transcription quantitative PCR (RT-qPCR). For each gene, its transcriptome-based expression levels (FPKMs) are presented as a small heatmap above the corresponding bar plot. Different lowercase letters on bars indicate significant differences by LSD test at P-value < 0.05.


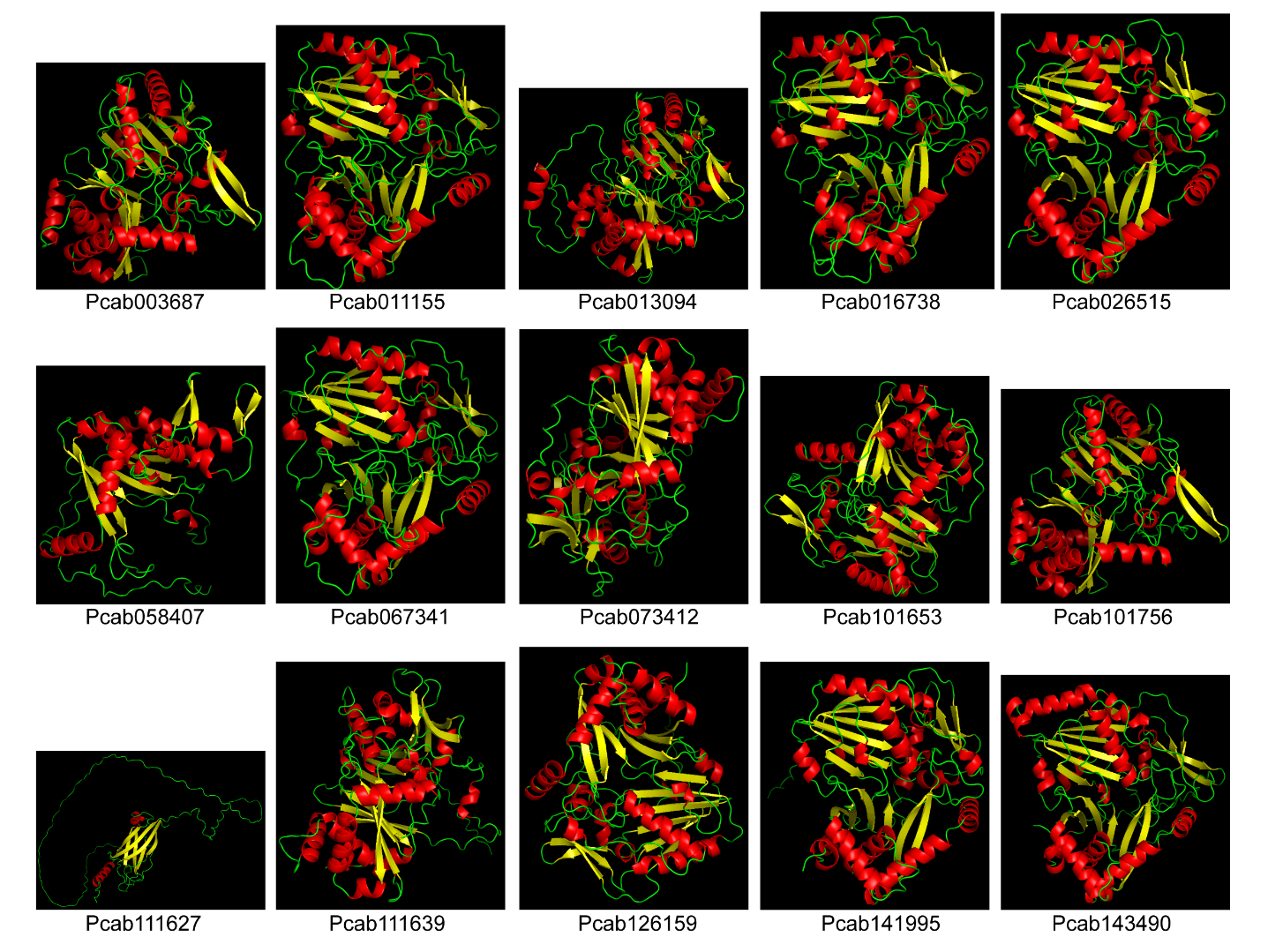


**Figure S7** Predicted protein structure of 15 potential BAHD-DCR acyltransferases of patchouli. The α-helixes, β-sheets, and unstructured loops of each protein are shown in red, yellow, and green color, respectively.
